# Genome sequence of the broad-host-range phage phi1_092033 against *Acinetobacter baumannii*

**DOI:** 10.1128/mra.00062-25

**Published:** 2025-03-25

**Authors:** Jiayuan Qin, Li Wei, Yu Feng, Zhiyong Zong

**Affiliations:** 1 Center of Infectious Diseases, West China Hospital, Sichuan University 12530, Chengdu, Sichuan, China; 2 Department of Infection Control, West China Hospital, Sichuan University 12530, Chengdu, Sichuan, China; 3 Division of Infectious Diseases, State Key Laboratory of Biotherapy 429364, Chengdu, Sichuan, China; 4 Center for Pathogen Research, West China Hospital, Sichuan University 12530, Chengdu, Sichuan, China; Portland State University, Portland, Oregon, USA

**Keywords:** *Acinetobacter baumannii*, carbapenem resistance, phage, phage therapy

## Abstract

We report the genome of a phage phi1_092033, isolated from sewage, which effectively lyses carbapenem-resistant *Acinetobacter baumannii* strains of various capsule types. phi1_092033 represents a species of genus *Saclayvirus*. Its genome consists of 104,070 bp, with a GC content of 38%, containing 188 protein-coding sequences and 13 tRNAs.

## ANNOUNCEMENT

Carbapenem-resistant *Acinetobacter baumannii* (CRAb) causes over 300,000 deaths globally each year (1). It is categorized by the World Health Organization (WHO) as a critical pathogen of concern (2). Treatment options for CRAb are extremely limited (3, 4), and phage therapy is a promising alternative (5). However, phages are typically narrow host range, which limits their applicability (6, 7). We present the genome of phi1_092033, a phage of the genus *Saclayvirus* that effectively lyses multiple capsule types of sequence type 2 (ST2) CRAb clinical isolates.

In January 2024, phi1_092033 was isolated from untreated sewage at the wastewater treatment station of West China Hospital. Phage enrichment was performed using the sputum-derived CRAb isolate 090908 as described previously (8). The study was approved by the Ethical Committee of West China Hospital. Single phage plaques measured 0.1–0.3 cm in diameter, were translucent, and were purified three times to ensure purity. Genomic DNA of phage phi1_092033 was extracted from purified phage particles using the Phage DNA Isolation Kit (Norgen Biotek, Thorold, Canada) according to the manufacturer’s instructions. Sequencing libraries were prepared using the NEBNext Ultra II DNA Library Prep Kit (New England Biolabs, Ipswich, MA), and sequencing was performed on the Illumina NovaSeq 6000 platform (Illumina, San Diego, CA). Raw sequencing reads underwent quality control with Trimmomatic v0.39 (9) to remove adapter sequences and discard reads shorter than 130 bases. Genome assembly was conducted with Unicycler v0.5.0 (10). Contamination screening and assembly completeness were assessed with CheckV (11), and non-phage contigs were removed prior to downstream analysis. The phage genome was annotated using Pharokka v1.7.2 (12). Antimicrobial resistance and virulence genes in the phi1_092033 genome were analyzed using the CARD (13) and VFDB (14) databases. The lifestyle of phi1_092033 was predicted using BACPHLIP (15). BLAST was used to search for phages sharing the highest overall DNA similarity (identity × coverage) with phi1_092033 (16).

**Fig 1:**
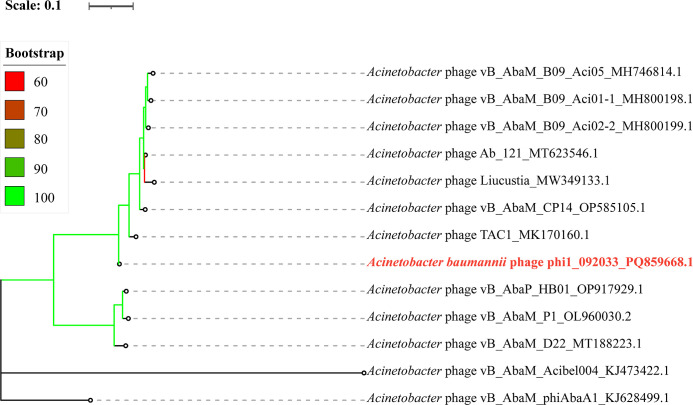
Phylogenetic tree of *Saclayvirus* phages. The maximum likelihood tree was constructed from an alignment of orthologous genes using IQ-TREE v2.3.6 under the GTR + F + I + G model with 1,000 ultra-fast bootstraps and was annotated using iTOL v6.9.

The genome sequencing of phi1_092033 produced 3,923,900 pairs of 150  bp reads, totaling 1.18 Gb (SRR31935446). After quality control, 3,908,664 reads remained, with a sequence coverage of 11,267×. The phi1_092033 genome has a length of 104,070 bp, a G + C content of 38%, an *N50* value of 104,070 bp, 188 predicted coding sequences (CDSs), and 13 tRNAs. The genome of phi1_092033 is complete (AAI_completeness value of 100) and free from contamination (contamination value of 0). No known antimicrobial resistance or virulence genes were identified. Furthermore, no lysogeny genes were detected, confirming its lytic nature. This was further supported by BACPHLIP analysis, which yielded a high BACPHLIP score of 99.95%. The BLAST results indicate that the genome of phi1_092033 exhibits the highest overall DNA similarity (89.65%, identity × coverage) to *Acinetobacter* phage TAC1 (accession no. MK170160.1), which belongs to the genus *Saclayvirus* in the class *Caudoviricetes*. Notably, the 89.65% overall DNA similarity is below the 95% threshold for species definition within the genus *Saclayvirus*. We clustered orthologous genes using PIRATE v1.0.5 (17) and aligned genes present in all genomes using MAFFT v7.526 (18). We inferred a maximum likelihood phylogenetic tree based on 46 core orthologous genes using IQ-TREE v2.3.6 (19). The resulting phylogeny placed phi1_092033 within a clade of *Saclayvirus* phages (Fig. 1). The above analyses indicate that phi1_092033 represents a novel species within the genus *Saclayvirus*.

We also characterized phi1_092033 for its host range using methods described previously (20, 21). Moreover, phi1_092033 was able to lyse ST2 CRAb isolates of capsule types KL2, KL7, KL77, KL101, KL160, and KL164. These results suggest that phi1_092033 holds significant potential as a therapeutic agent for CRAb infections and adds an important member to the phage therapeutic arsenal.

## Data Availability

The complete genome sequence of *Acinetobacter baumannii* phage phi1_092033 has been deposited in GenBank under accession number PQ859668.1 and Sequence Read Archive (SRA) accession number SRR31935446. The version described in this paper is the first version.

## References

[1] Antimicrobial Resistance Collaborators. 2022. Global burden of bacterial antimicrobial resistance in 2019: a systematic analysis. Lancet 399:629–655. doi:10.1016/S0140-6736(21)02724-0 35065702 PMC8841637

[2] World Health Organization. 2024. WHO Bacterial Priority Pathogens List, 2024: Bacterial Pathogens of Public Health Importance to Guide Research, Development and Strategies to Prevent and Control Antimicrobial Resistance. Available from: https://www.who.int/news/item/27-02-2017-who-publishes-list-of-bacteria-for-which-new-antibiotics-are-urgently-needed

[3] Iovleva A, Fowler VG, Doi Y. 2025. Treatment approaches for carbapenem-resistant Acinetobacter baumannii Infections. Drugs 85:21–40. doi:10.1007/s40265-024-02104-6 39607595 PMC11950131

[4] Shields RK, Paterson DL, Tamma PD. 2023. Navigating available treatment options for carbapenem-resistant Acinetobacter baumannii-calcoaceticus complex infections.. Clin Infect Dis 76:S179–S193. doi:10.1093/cid/ciad094 37125467 PMC10150276

[5] Koncz M, Stirling T, Hadj Mehdi H, Méhi O, Eszenyi B, Asbóth A, Apjok G, Tóth Á, Orosz L, Vásárhelyi BM, et al.. 2024. Genomic surveillance as a scalable framework for precision phage therapy against antibiotic-resistant pathogens. Cell 187:5901–5918. doi:10.1016/j.cell.2024.09.009 39332413

[6] Oechslin F. 2018. Resistance development to bacteriophages occurring during bacteriophage therapy. Viruses 10:351. doi:10.3390/v10070351 29966329 PMC6070868

[7] Egido JE, Costa AR, Aparicio-Maldonado C, Haas PJ, Brouns SJJ. 2022. Mechanisms and clinical importance of bacteriophage resistance. FEMS Microbiol Rev 46:fuab048. doi:10.1093/femsre/fuab048 34558600 PMC8829019

[8] Li J, Fang Q, Luo H, Feng Y, Feng Y, Zong Z. 2025. “Sichuanvirus”, a novel bacteriophage viral genus, able to lyse carbapenem-resistant Klebsiella pneumoniae. BMC Microbiol 25:17. doi:10.1186/s12866-024-03736-0 39806322 PMC11726925

[9] Bolger AM, Lohse M, Usadel B. 2014. Trimmomatic: a flexible trimmer for Illumina sequence data. Bioinformatics 30:2114–2120. doi:10.1093/bioinformatics/btu170 24695404 PMC4103590

[10] Wick RR, Judd LM, Gorrie CL, Holt KE. 2017. Unicycler: resolving bacterial genome assemblies from short and long sequencing reads. PLoS Comput Biol 13:e1005595. doi:10.1371/journal.pcbi.1005595 28594827 PMC5481147

[11] Nayfach S, Camargo AP, Schulz F, Eloe-Fadrosh E, Roux S, Kyrpides NC. 2021. CheckV assesses the quality and completeness of metagenome-assembled viral genomes. Nat Biotechnol 39:578–585. doi:10.1038/s41587-020-00774-7 33349699 PMC8116208

[12] Bouras G, Nepal R, Houtak G, Psaltis AJ, Wormald PJ, Vreugde S. 2023. Pharokka: a fast scalable bacteriophage annotation tool. Bioinformatics 39:btac776. doi:10.1093/bioinformatics/btac776 36453861 PMC9805569

[13] Alcock BP, Raphenya AR, Lau TTY, Tsang KK, Bouchard M, Edalatmand A, Huynh W, Nguyen AV, Cheng AA, Liu S, et al.. 2020 CARD 2020: antibiotic resistome surveillance with the comprehensive antibiotic resistance database. doi:10.1093/nar/gkz935 PMC7145624 31665441

[14] Chen L, Yang J, Yu J, Yao Z, Sun L, Shen Y, Jin Q. 2005. VFDB: a reference database for bacterial virulence factors. Nucleic Acids Res 33:D325–8. doi:10.1093/nar/gki008 15608208 PMC539962

[15] Hockenberry AJ, Wilke CO. 2021. BACPHLIP: predicting bacteriophage lifestyle from conserved protein domains. PeerJ 9:e11396. doi:10.7717/peerj.11396 33996289 PMC8106911

[16] Altschul SF, Gish W, Miller W, Myers EW, Lipman DJ. 1990. Basic local alignment search tool. J Mol Biol 215:403–410. doi:10.1016/S0022-2836(05)80360-2 2231712

[17] Bayliss SC, Thorpe HA, Coyle NM, Sheppard SK, Feil EJ. 2019. PIRATE: A fast and scalable pangenomics toolbox for clustering diverged orthologues in bacteria. Gigascience 8:giz119. doi:10.1093/gigascience/giz119 31598686 PMC6785682

[18] Katoh K, Misawa K, Kuma K, Miyata T. 2002. MAFFT: a novel method for rapid multiple sequence alignment based on fast Fourier transform. Nucleic Acids Res 30:3059–3066. doi:10.1093/nar/gkf436 12136088 PMC135756

[19] Minh BQ, Schmidt HA, Chernomor O, Schrempf D, Woodhams MD, von Haeseler A, Lanfear R. 2020. IQ-TREE 2: new models and efficient methods for phylogenetic inference in the genomic era. Mol Biol Evol 37:1530–1534. doi:10.1093/molbev/msaa015 32011700 PMC7182206

[20] Yin X, Zong Z. 2022. Safety and efficacy of phage therapy in difficult-to-treat infections: the taxonomic concern. Roum Arch Microbiol Immunol 82:16–24. doi:10.54044/RAMI.2023.01.03

[21] Fang Q, Feng Y, McNally A, Zong Z. 2022. Characterization of phage resistance and phages capable of intestinal decolonization of carbapenem-resistant Klebsiella pneumoniae in mice. Commun Biol 5:48. doi:10.1038/s42003-022-03001-y 35027665 PMC8758719

